# Rescue therapy with an albuvirtide-based antiretroviral regimen in an HIV-infected child with multidrug resistance and multiple opportunistic infections: a case report

**DOI:** 10.1186/s12981-023-00560-w

**Published:** 2023-08-28

**Authors:** Wei Tang, Xiao-yun Song, Jing Cao, Chun Liu, Fang Zheng

**Affiliations:** The First Hospital of Changsha, Changsha, Hunan People’s Republic of China

**Keywords:** HIV-child, AIDS, Albuvirtide, Drug resistance, Case report

## Abstract

**Background:**

Managing multidrug-resistant (MDR) HIV infections in children is particularly challenging due to the lack of experience with new drugs in the pediatric setting. Second-line albuvirtide (ABT) with an optimized antiretroviral background therapy was approved for adults and adolescents after first-line treatment failure. This paper describes the treatment outcomes and adverse effects of an ABT-based dual-active antiretroviral treatment regimen in a child with MDR HIV strains.

**Case presentation:**

A 13 year-old Chinese female patient infected with MDR HIV strains showed a decrease in viral load (from 4.48 log10 to 1.73 log10) and an increase in CD4 + T cells (from 15 to 308 cells/µl) after 12 months of treatment with an ABT-based antiretroviral regimen. The child showed no relevant drug-related adverse reactions.

**Conclusions:**

The case reported here could suggest that an ABT-based antiretroviral therapy might be beneficial and without relevant toxicity in children with MDR HIV. Infectiologists specializing in managing HIV should be prepared to manage an increasing number of children with MDR HIV. ABT might be a new treatment option for MDR HIV infection in children.

## Background

HIV/AIDS is a major public health concern, with 38.0 million people living with HIV infection in 2020, including 2.78 million children (< 19 years of age) [1]. Fortunately, improvements in the access to services for preventing mother-to-child transmission (MTCT) of HIV and antiretroviral therapy (ART) led to reductions of 20% and 50% in the numbers of AIDS-related deaths and new HIV infections among children [2].

Children with HIV will require life-long and long-term therapy, increasing the risk of developing HIV drug resistance (HIVDR) due to inadequate drug compliance, the lack of evidence-based data regarding specific ART dosing in different age groups, and suboptimal regimens or plasma drug levels. Children have higher rates of HIVDR than adults with heterosexually transmitted HIV and have a 2.2-fold higher risk of virological failure (VF) than adults after 5 years of ART [3].

Albuvirtide (ABT) is a long-acting injectable ART approved by the Chinese National Medical Products Administration (NMPA) in 2018 and has broad-spectrum anti-HIV-1 activity in vitro, with a half-maximal inhibitory concentration (IC50) of 0.5-5.0 nmol/l.

Herein, we report a 13-year-old Chinese female child with AIDS and MDR and the treatment effect and adverse effects of an ABT-based active antiretroviral treatment regimen that is the first case report in the world.

## Case presentation

A 13 year-old female child with HIV progressed to AIDS despite treatments and was admitted to the First Hospital in Changsha in June 2020. She was infected through MTCT, and her parents both died of AIDS. At admission, her legal guardians reported persistent cough with white sputum, nausea, vomiting, and weight loss for 4 months.

The patient was infected with HIV at birth, and her parents died of AIDS many years ago. She was diagnosed with HIV infection at 1 year of age but started ART at 6 years old. The initial ART regimen was unclear before 2016, till she started the 3TC/ABC/LPV/r regimen.

At admission, the viral load and CD4 + T-cell counts were 6.16 log10 copies/mL and 2 cells/µl, respectively. AIDS, along with an opportunistic infection, was diagnosed. Sulfanilamide was given against *Pneumocystis pneumonia* (PCP), and fluconazole was given as an empirical antifungal treatment. She continued the previous ART regimen of 3TC/ABC/LPV/r. The patient recovered and was discharged after 20 days of treatment.

**Fig. 1:**
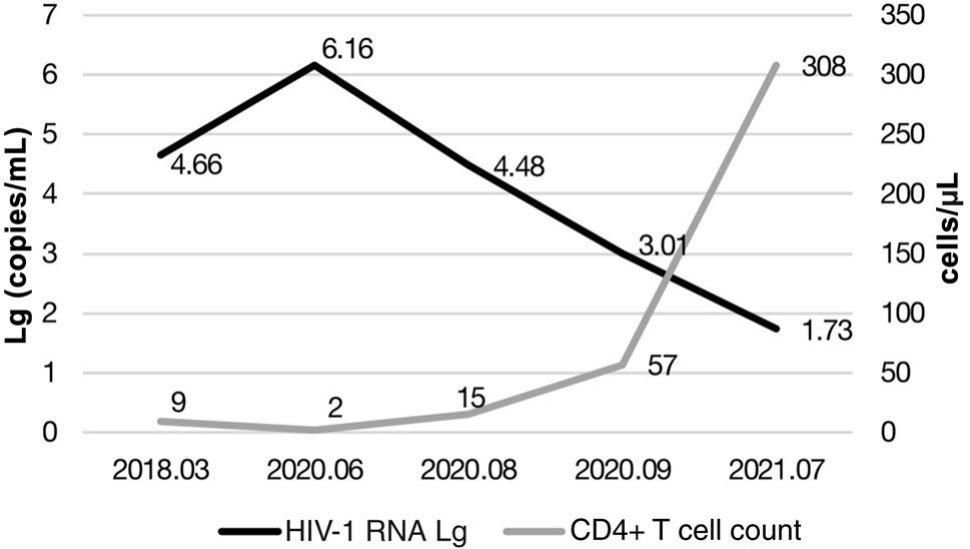
CD4 + cell counts and HIV RNA evolution during follow-up

In August 2020, the viral load and CD4 + T cell counts were 4.48 log10 copies/mL and 15 cells/µl, respectively, and an MDR test (Sanger sequencing) detected 15 point mutations in the reverse transcriptase (M41L, E44A, F77L, M184V, L210W, T215Y, K219N, K101H, and Y181C) and protease (M46I, L76V, 184 V, K20T, Q58E, and G73S) (Table 1). Then, an adjusted ART regimen of ABT (160 mg, 1 weekly) with dolutegravir (DTG, 50 mg, daily) was given. After 1 month, the HIV viral load was decreased to 3.01 log10 copies/mL, and the CD4 + T-cell count was increased to 57 cells/µl (Fig. 1).

**Table 1:** Genotypic drug resistance interpretation according to the HIV drug resistance database system

				June 2020	July 2021
Gene coding regions	Mutations		ARV drugs		Interpretation ^a^
PR	M 46I, L76V, 184 V, K20T, Q58E, G73S		Atazanavir (ATV/r)	H	H
			Darunavir (DRV/r)		I
			Fosamprenavir (FPV/r)		H
			Indinavir (IDV/r)		H
			Lopinavir (LPV/r)	H	H
			Nelfinavir (NFV)		H
			Saquinavir (SQV/r)		H
			Tipranavir (TPV/r)		I
RT	NRTI relevant	M41L, E 44 A, F77L, M184V, L210W, T215Y, K219N	Abacavir (ABC)	H	H
			Zidovudine (AZT)	H	H
			Stavudine (d4T)		H
			Didanosine (DDI)		H
			Emtricitabine (FTC)	H	H
			Lamivudine (3TC)	H	H
			Tenofovir (TDF)	H	H
	NNRTI relevant	K101H, Y181C	Doravirine (DOR)		P
			Efavirenz (EFV)	I	I
			Etravirine (ETR)	I	I
			Nevirapine (NVP)	H	H
			Rilpivirine (RPV)	I	I
IN	INSTI relevant		Bictegravir (BIC)	S	S
			Cabotegravir (CAB)		S
			Dolutegravir (DTG)	S	S
			Elvitegravir (EVG)	S	S
			Raltegravir (RAL)	S	S

*PR* protease region, *RT* reverse transcriptase region, *ARV* anti-retroviral

^a^ Interpretation: *S* susceptible, *I* intermediate, *H* highly resistant, *P* potential resistant

In July 2021, the HIV viral load was decreased to 1.73 log10 copies/mL, and the CD4 + T-cell count was increased to 308 cells/µl (Fig. 1). Moreover, this ART regimen was well tolerated, without relevant toxicity, and the patient’s condition improved greatly.

The patient ultimately developed classical Hodgkin lymphoma in January 2022. Unfortunately, due to the foster family’s economic condition, poor family care, medication non-compliance, and a low desire for life, the patient died on March 5, 2022. The main cause of death was Hodgkin lymphoma.

## Discussion and conclusions

Children infected with HIV via vertical transmission will eventually develop significant HIV MDR due to low doses of non-suppressive regimens and/or inadequate ART adherence [3]. Data from the World Health Organization (WHO) demonstrated that five in every 10 young children diagnosed with HIV harbor resistant HIV [4]. The drug resistance rates in children failing first- and second-line treatments are 34–99%, and the resistance primarily consists of resistance to non-nucleoside reverse transcriptase inhibitors (NNRTI) and the nucleoside reverse transcriptase inhibitors (NRTI) mutation M184V, limiting the treatment options, especially in resource-limited settings [2]. Therefore, it is crucial to describe more suitable first- or second-line therapies for children with HIV MDR.

The patient reported here was infected through MTCT, and her parents died of AIDS. She had an incomplete adherence to ART, and the viral load was very high with low CD4 + T cell counts. Therefore, the likelihood of developing MDR was high.

ABT is a long-acting HIV fusion inhibitor used in patients > 16 years of age. It is active against 28 different clinical isolates of HIV-1 in China, with IC50 values ranging from 1.3 to 18.1 nmol/L [5]. Despite good efficacy, safety, and tolerability in adults, the effects of ABT in children have not been documented. ABT exhibits activity in the presence of enfuvirtide (T20)-associated mutations due to its nature of genetic barrier [5]. In a phase III trial, ABT combined with LPV/r was proven non-inferior to the WHO-recommended three-drug second-line therapy in treatment-experienced HIV patients with MDR. The study reported that 75.7% of patients treated with ABT plus LPV/r achieved HIV-1 RNA levels < 50 copies/mL after 48 weeks [6].

Since DTG is effective in children > 4 weeks of age and weighing > 3 kg, the WHO recommends the ABC + 3TC + DTG combination as the preferred first-line regimen for children. Nevertheless, DTG monotherapy was associated with an increased risk of virological failure and DTG resistance in adults. Furthermore, the case reported here showed MDR to protease inhibitors, NRTIs, and NNRTIs. Therefore, a combination of ABT and DTG was used, the viral load was decreased, and the CD4 + T cells count was increased after 4 weeks. After 1 year, the viral load was maintained at low levels. Before switching to an ABT-based regimen, the child demonstrated a depletion of CD4 + T cells, but CD4 + T-cell recovery to 308 cells/µl was observed after ABT + DTG treatment. A sufficient CD4 + response with ART was evidenced as 50–150 cells/µl [7].

In the patient reported here, the HIV RNA was 54 copies/mL but did not achieve < 20 copies/mL virologic suppression. High pre-treatment HIV-RNA levels are an independent predictor of delayed virological suppression and increased risk of virological failure [8, 9]. Unfortunately, this child developed and died of classical Hodgkin lymphoma. Nonetheless, an ABT-based regimen demonstrated a good effectiveness and safety profile during the 1 year of treatment. No injection site reaction and ABT-related adverse events were observed.

In conclusion, this is the first reported case of the use of ABT in a child with virological failure. Furthermore, it provided information on the use of ABT in a child with MDR HIV and showed good effectiveness and safety for nearly 1 year.

## Data Availability

The datasets used and/or analysed during the current study are available from the corresponding author on reasonable request.

## Abbreviations

ABT: Albuvirtide
ART: Antiretroviral therapy
ARV: Anti-retroviral
DTG: Dolutegravir
HIVDR: HIV drug resistance
MDR: Multidrug-resistant
MTCT: Mother-to-child transmission
NMPA: National medical products administration
NNRTI: Non-nucleoside reverse transcriptase inhibitors
NRTI: Nucleoside reverse transcriptase inhibitors
PCP: Pneumocystis pneumonia
PR: Protease region
RT: Reverse transcriptase region
VF: Virological failure
WHO: World Health Organization
